# Healthcare professionals’ perspectives on facilitators of and barriers to CKD management in primary care: a qualitative study in Singapore clinics

**DOI:** 10.1186/s12913-022-07949-9

**Published:** 2022-04-26

**Authors:** Chandrika Ramakrishnan, Ngiap Chuan Tan, Sungwon Yoon, Sun Joon Hwang, Marjorie Wai Yin Foo, Muthulakshmi Paulpandi, Shi Ying Gun, Jia Ying Lee, Zi Ying Chang, Tazeen H. Jafar

**Affiliations:** 1 Duke-NUS Medical School, Program in Health Services & Systems Research, 8 College Road Singapore 169857, Singapore, Singapore; 2 Department of Research, SingHealth Polyclinics, Singapore, Singapore; 3 General Practice, SingHealth Polyclinics, Singapore, Singapore; 4 Department of Renal Medicine, Singapore General Hospital, Singapore, Singapore

**Keywords:** CKD, Healthcare professionals, Polyclinics, Barriers, Facilitators, Primary care, TDF

## Abstract

**Introduction:**

The burden of chronic kidney disease (CKD) is rising globally including in Singapore. Primary care is the first point of contact for most patients with early stages of CKD. However, several barriers to optimal CKD management exist. Knowing healthcare professionals’ (HCPs) perspectives is important to understand how best to strengthen CKD services in the primary care setting. Integrating a theory-based framework, we explored HCPs’ perspectives on the facilitators of and barriers to CKD management in primary care clinics in Singapore.

**Methods:**

In-depth interviews were conducted on a purposive sample of 20 HCPs including 13 physicians, 2 nurses and 1 pharmacist from three public primary care polyclinics, and 4 nephrologists from one referral hospital. Interviews were audio recorded, transcribed verbatim and thematically analyzed underpinned by the Theoretical Domains Framework (TDF) version 2.

**Results:**

Numerous facilitators of and barriers to CKD management identified. HCPs perceived insufficient attention is given to CKD in primary care and highlighted several barriers including knowledge and practice gaps, ineffective CKD diagnosis disclosure, limitations in decision-making for nephrology referrals, consultation time, suboptimal care coordination, and lack of CKD awareness and self-management skills among patients. Nevertheless, intensive CKD training of primary care physicians, structured CKD-care pathways, multidisciplinary team-based care, and prioritizing nephrology referrals with risk-based assessment were key facilitators. Participants underscored the importance of improving awareness and self-management skills among patients. Primary care providers expressed willingness to manage early-stage CKD as a collaborative care model with nephrologists. Our findings provide valuable insights to design targeted interventions to enhance CKD management in primary care in Singapore that may be relevant to other countries.

**Conclusions:**

The are several roadblocks to improving CKD management in primary care settings warranting urgent attention. Foremost, CKD deserves greater priority from HCPs and health planners. Multipronged approaches should urgently address gaps in care coordination, patient-physician communication, and knowledge. Strategies could focus on intensive CKD-oriented training for primary care physicians and building novel team-based care models integrating structured CKD management, risk-based nephrology referrals coupled with education and motivational counseling for patients. Such concerted efforts are likely to improve outcomes of patients with CKD and reduce the ESKD burden.

**Supplementary Information:**

The online version contains supplementary material available at 10.1186/s12913-022-07949-9.

## Introduction

Chronic kidney disease (CKD), with a worldwide prevalence of 11%-13%, is an increasingly common public health problem [1]. CKD is associated with premature cardiovascular disease, all-cause mortality, and high risk of progression to end-stage kidney disease (ESKD) requiring costly kidney replacement therapy- dialysis or kidney transplant [2, 3]. CKD is one of the steepest rising causes of death globally, including in Singapore, which ranks fifth worse in incident ESKD worldwide [4, 5].

Kidney Disease Improving Global Outcome (KDIGO) classifies patients with CKD into categories according to level of estimated glomerular filtration rate (eGFR) and albuminuria based on the risk of progression to ESKD [6]. Most patients with CKD are usually asymptomatic until the disease becomes advanced, i.e., eGFR < 30 ml/min/1.73m^2^. Primary care is the first point of contact of most patients with early stages of CKD with the health system worldwide and in Singapore. Prompt institution of non-pharmacologic and pharmacologic therapy at early stages of CKD can preserve kidney function and prevent CVD [7]. However, serious gaps exist in clinical practices with regards to management of CKD. In an analysis of 20,538 individuals in the US, 50% of patients with proteinuric CKD were not receiving the recommended renin–angiotensin–aldosterone system (RAAS) blockers [8]. Likewise in Singapore, less than 50% of patients with diabetes and CKD had blood pressure control to < 140/90 mm Hg, and less than 50% had glycated hemoglobin of < 7% [9].

Existing literature suggests impediments to CKD management in primary care exists at multiple levels [10]. Patient-reported challenges and perceptions on CKD management are well documented [11]. However, there is limited understanding on the challenges and opportunities to enhance CKD management from perspectives of HCPs involved in direct care of patients with CKD. Including HCPs from primary care and nephrologists, key stakeholders in the CKD care spectrum, will maximize the diversity of perceptions and opinions on CKD management. Underpinned by the Theoretical Domains Framework (TDF), the study aimed to explore HCPs’ perspectives on facilitators of and barriers to CKD management in primary care in Singapore.

## Methods

### Study setting and design

Primary healthcare for the multi-ethnic population in Singapore is delivered as a fee-for-service via a network of public sector primary care polyclinics and private general practitioner clinics, the former providing care to the majority of patients with chronic diseases [12]. The public polyclinics are well equipped “one-stop” centers and offer comprehensive range of health care services at markedly subsidized rates. Primary care polyclinic model includes referrals to the specialists at the affiliated hospitals for additional evaluation and risk assessment for chronic conditions (eg CKD, cardiovascular diseases). After specialist evaluation patients are often referred back to polyclinics for further management with recommendations from specialists, and often patients are co-managed by primary care physicians with periodic 6-monthly or yearly visits to the specialists.

The study set in SingHealth, a large healthcare cluster with a network of nine public polyclinics and three referral hospitals included a sampling frame of multidisciplinary stakeholders (physicians, nurses, and pharmacist) from three polyclinics with experience of managing patients with CKD in the past year, and nephrologists from the specialist outpatient clinic (SOC) at one affiliated referral hospital. In order to maximize the diversity in views and experiences, participants were recruited in terms of years in practice and practice location. A total of 21 potential participants were approached with an invitation letter through their respective department heads, and except for one participant who did not agree due to time constraints, all others accepted to attend the interview. Participants were contacted by the study coordinator thorough email and interviews were scheduled.

### Conceptual framework

We used the Theoretical Domains Framework (TDF) version 2 to guide the interviews and analyze data. TDF is a single framework that synthesizes 128 constructs from 33 behavior and behavioral change theories clustered into 14 domains [13, 14]. TDF has capacity to elicit a comprehensive set of beliefs and is informative regarding the potential mediators of behavior change. Our interview probes adapted the TDF framework to discern barriers and facilitators regarding HCP’s perception to eventually develop effective interventions to support optimal CKD management in primary care [15].

### Data collection

In-depth interviews were conducted on a purposive sample of 20 participants comprising 13 physicians, 4 nephrologists, 2 nurses and 1 pharmacist. A semi-structured interview guide with open-ended questions developed by qualitative research and implementation science experts explored participants’ perceived knowledge and skills in CKD management, professional role, confidence in managing CKD, ability to reveal CKD diagnosis, communication capabilities, barriers and facilitators to CKD management, decision process for referrals and usefulness of decision-support tools. Pre-testing was undertaken on three individuals and their responses was used to refine the interview questions. All interviews took place in private consultation rooms in the healthcare facility to ensure participant comfort and lasted between 30–60 min. The interviews were conducted in English by the research team members (TNC, CR, SY, PM) trained in qualitative methodologies and extensive experience conducting in-depth interviews. The participants had no personal or professional relationship with the interviewers, and this allowed participants to express their opinions freely. Interviews were audio recorded and transcribed verbatim by research team members.

### Ethical approval

The SingHealth Centralized Institutional Review Board (CIRB) approved the study. Prior to interviews, participants signed a written informed consent for participation, for audio recording of interviews and confidentiality of their responses. All methods were performed in accordance with relevant guidelines and regulations.

### Data analysis

Verbatim transcripts of the interviews were reviewed and checked with the audio recordings for accuracy. Two researchers (CR, SJH) independently conducted the data analysis using QSR NVivo 11 software. Data was analyzed using both inductive and deductive methods [16]. At first, the researchers read and re-read the transcripts and familiarized with the data. The thematic analytic process involved coding, repetitive sorting and comparison. Each transcript was open-coded line by line to create code components. The code components were compared and grouped into categories and themes pertaining to facilitators and barriers to CKD management. The categories and themes were subsequently mapped against the relevant domains within the TDF. Domains with overlapping and inter-linked themes that reflected key clinical behaviors and tasks relevant to CKD management were combined to represent meaningful concepts to systematically understand barriers and facilitators. For example, when themes such as knowledge gaps in junior doctors or lack of self-management skills in patients were identified, they were assigned to the TDF domain of ‘knowledge & skills.’ Consensus meetings were held to ensure that themes were mutually exclusive, and that mapping reflected an accurate conceptualization of the TDF domains. Discrepancies were resolved and deviant cases identified. All themes were aligned with the domains within TDF. Thematic saturation was achieved after analyzing 15 transcripts. We anchored the methodology with reference to COREQ [17]. (See additional file 1 COREQ checklist).

## Results

In all, 20 participants completed the interviews of which 14 (70%) were females and the mean age was 37.7 years. The participants comprised of 13 (65%) physicians, 4 (20%) nephrologists, 2 (10%), nurses and 1 (5%) pharmacist. The mean years of service was 9.3 years, and 10 (50%) participants had more than 10 years of professional experience. Most participants (90%) managed 31 or more patients with CKD in the past year. Table 1 summarizes the participant characteristics.

**Table 1:** Characteristics of participants

Characteristics *n* = 20	
**Sex**	**n (%)**
Males	6 (30)
Females	14 (70)
**Age, mean years (SD)**	**37.7(8.2)**
**Age distribution**	
21–30 years	2
31–40 years	13
41–50 years	2
51–60 years	3
**Profession /role n (%)**	
Physician	13 (65)
Nephrologist	4 (20)
Nurse	2 (10)
Pharmacist	1 (5)
**No of CKD patients managed in the past year n (%)**	
1–10	1(5)
11–20	1(5)
21–30	0
31 or more	18 (90)
**Years in service, mean years (SD) 9.3 (5.6)**	
**Years in service**	**n (%)**
< 5 years	4 (20)
5–10 years	6 (30)
> 10 years	10 (50)
**Years in service based on profession/role**	**Mean years**
Physicians	7.1
Nephrologists	13
Nurses	17
Pharmacist	7

**Fig. 1:**
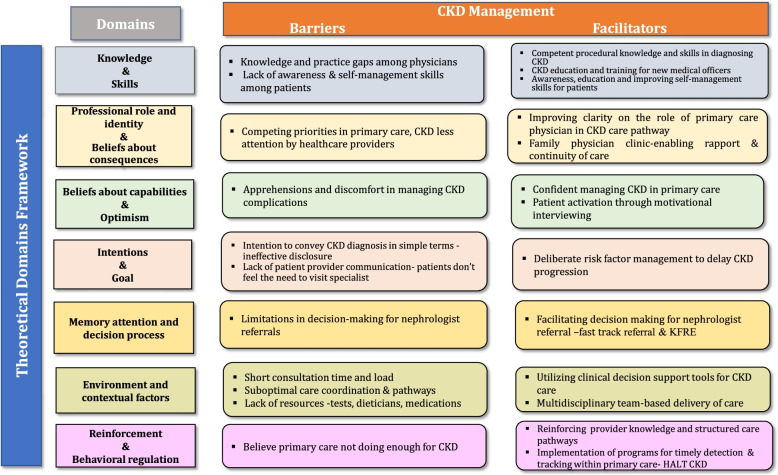
Conceptualizing barriers and facilitators to CKD management using the Theoretical Domains Framework Version 2

HCPs’ perceived barriers and facilitators to CKD management were captured under themes encompassing 12 of the 14 TDF domains. Overall, 24 themes including 11 barriers and 13 facilitators to CKD management emerged and described under relevant TDF domains. Figure 1 illustrates the barriers and facilitators to CKD management conceptualized using the TDF version 2.

### Barriers to CKD management

#### Perceived knowledge and skills

One of the key barriers to CKD management was differential level of competency among physicians, with few openly accepting knowledge and practice gaps on CKD management. Some physicians perceived limited information on CKD management in their reference handbooks and felt challenged due to lack of protocols or clinical practice guidelines similar to diabetes. As a result, physicians felt unprepared, and attributed the shortcomings to lack of CKD training as an important barrier.

“I think, even our new medical officers who are coming in, if I remember correctly, the induction program didn’t cover anything on renal, I think, if I’m not wrong – mainly just hypertension and common things.” Participant 14_Physician.

Several providers discussed the lack of CKD awareness and self-management skills among patients as a barrier to CKD management. Over 50% of patients, especially in earlier stages of CKD, i.e. CKD stage 3, and the majority of elderly, were unaware of their CKD status.

“May be if I have to put a number to it, may be about at least more than 50 percent not aware especially those with milder CKD…like stage 3’’ Participant 06_Physician.

Participants recounted poor health knowledge, variable level of awareness, poor self-management skills with lack of sustained behavioral modifications and issues with medication adherence among patients with CKD that necessitated a more paternalistic approach from physicians. However, few physicians had divergent views and believed that younger patients and individuals with CKD stage 4 were often aware of their condition, and often times fear of dialysis motivated and nudged them to be adherent to management recommendations.

#### Professional role and identity & beliefs about consequences

HCPs in primary care believed their role was important as the key caregiver for patients with chronic conditions. Although they prioritized managing chronic diseases like diabetes, hypertension, and hyperlipidemia, CKD was not a priority. They felt that since CKD was primarily asymptomatic, it is often neglected during clinical consultations. Perceptions on prioritizing CKD in primary care were mixed. Some respondents perceived CKD was 'not viewed in the same league as diabetes, stroke, or heart attacks'. In contrast, some respondents stressed the need to consider CKD as an essential condition needing more attention in primary care. Nonetheless, physicians expressed that due to competing priorities in a consultation, especially for patients with multiple morbidities, CKD received less attention in primary care.

“It is just that we don’t routinely look into the CKD and our knowledge on treating patients with CKD is really lacking. So therefore, currently we give very little attention to CKD.” Participant 05_Physician.

#### Belief about capabilities & optimism

Although respondents strongly believed in their capabilities for treating general aspects of CKD, some physicians expressed apprehensions and felt unfamiliar in prescribing higher doses of blood pressure medications, managing hyperkalemia, and advising kidney diet. More often, such precarious situations prompted their referrals to nephrologists.

“I think the most difficult one is when does it come to a point where we need to stop medicines or adjust the renal medicines or the renal doses. For things that, you know, usually when managing protein urea and all that, usually in the outpatient clinic setting, we only have a maximum dose you can give. So, and also, the experience that we have is, I mean, quite limited I would say, so we don’t really increase the medicines although we can.” Participant 01_Physician.

#### Intentions and goals

With an intention not to cause alarm or create anxiety for patients, many physicians did not proactively bring up CKD during consultations.

“So, most of the time, we’ll pick it up quite early because of the screening, so most of the patients don’t have the CKD. And then when they start, they do get the CKD, maybe, like stage 2, we won’t really alarm them” Participant 14_Physician.

When physicians discussed CKD, it was common to use simple language and ‘lay terms’ like ‘weak kidneys’ but refrained from explaining CKD stages or severity. Participants highlighted that physician–patient communication was not always optimal and led to ineffective CKD diagnosis disclosure. One participant expressed that often patients are astonished when physicians reveal CKD diagnosis.

“They (patients) are very surprised, and they react as if they have not been told before, even though they may have had it for years.” Participant 06_Physician.

Nephrologists expressed concern over the ineffective CKD diagnosis disclosure in primary care citing numerous episodes of patients not comprehending the reason for their renal clinic referral. They also noted the patient’s reluctance for a nephrologist’s review. However, few physicians articulated their desire, intent, and efforts to communicate with patients but were confounded by patient-level challenges of poor CKD knowledge and awareness.

#### Memory attention and decision process

Polyclinics have standardized criteria for specialist referrals. Nevertheless, physicians expressed limitations in decision-making for specialist referrals due to ‘doubts’, ‘fear of missing out on things’, ‘to be seen early’ and ‘not wanting to wait'.

“So usually, I won’t wait for the patient to be severe and then I refer. But, I usually if it is stage 4, I will definitely offer a referral whether the patient take it themselves after the discussion”. Participant 06_Physician.

Although nephrologists expected most referrals in stage 3b, nevertheless they expressed that the “volume of referrals was tremendous”,. They felt that physicians in primary care were unable to adhere to explicit referral criteria which resulted in inappropriate referrals especially of elderly with mild reduction in eGFR thereby creating a burden on the renal specialist clinics. However, the nephrologists also recounted numerous instances of late referrals.

“It’s (referral) very standard. They have to fit the certain criteria before you can refer here (specialist clinic), so there are some tendencies, so the doctor that really wants to refer even though it doesn’t really fulfil the criteria, just anyhow click on all of them. So sometimes, we do encounter such situations, which is unavoidable. Sometimes they don’t read the criteria properly and they still refer**”.** Participant 18_Nephrologist.

#### Environmental context and resources

Environmental context and resources emerged as a key domain influencing CKD management. Participants identified a wide range of organizational factors like short consultation time, suboptimal care coordination and pathways, and lack of resources impeding CKD management. Most respondents expressed short consultation time is a key barrier to managing CKD. Participants found consultation duration of 5–10 min limiting for CKD to go ‘through in detail,’ and the need to address other conditions first, with CKD ‘just be seconded around and forgotten.’ In addition, most respondents perceived time constraints limiting discussions on CKD, often times impeding patient-physician communication.

Most respondents commonly mentioned long waiting time for specialist appointments, fragmentation of care, and obscure medical information exchange between primary care- nephrologists that together ensued suboptimal care coordination for CKD. In addition, almost all respondents complained about the long waiting time, with delays in securing specialist appointments needing a lead time of about 3–6 months to see a kidney specialist.

“I think the referral time for them (patients) to see, the kidney doctor can take a while. I think it [specialist appointment] is very long. I think there was a time it was, waiting time can be 6 months also.” Participant 04_Physician.

Adding to the delay, the system of rotation of physicians in the polyclinics led to fragmentation and lack of continuity of care.

“The one (thing) I wanted to mention was (about) the different doctors. So, that may play a factor because sometimes we are not the same doctor that keeps seeing the patient. So, (firstly), you may not have the doctor-patient relationship to be able to communicate that to the patient.” Participant 14_Physician.

Physicians coordinated specialist referrals and often relied on nephrologist’s feedback to follow-up on treatment recommendations. However, obscure medical information exchange between physicians and specialists had a profound impact on care coordination for CKD. Many participants expressed needing more time to screen through electronic documentation in the information systems for patients with multiple specialist visits. Many respondents narrated incidents of patients losing memos and expressed concerned on the suboptimal documentation and communication between physicians and specialists.

**“**Even though there is some kind of an electronic documentation it is still not optimal. That memo, that hard copy memo may not get to where it is. Now, do I email? No, I don’t email primary, I have no idea where to email.” Participant 19_Nephrologist.

Primary care did have most essential components for managing CKD. However, few respondents perceived lack of resources. Lack of access to laboratory tests like serum calcium and bicarbonate needed to monitor later stages of CKD, and non-availability of some combination anti-hypertensive agents that patients can potentially have their choices to were disclosed by some physicians. Few respondents desired nutrition counseling for patients with CKD, but the polyclinics were constrained on availability of dieticians and incurring additional costs for patients.

“For dietician we only have one dietician available in Singhealth polyclinic because for dietician there is a charge”. Participant 16_Nurse.

#### Reinforcement & behavioral regulation

Primary care management of early CKD is of utmost importance. However, some HCPs opined that they were just offering basic care at the polyclinics and not helping patients control their disease optimally. Given the increasing CKD burden, primary care needed to have a greater role in the care for CKD. However, few physicians felt otherwise and were concerned that primary care was not doing enough for CKD.

“I think with the increasing number of patients with CKD, the role is greater and but having said that we usually only manage the very milder cases. The more serious ones, the later stage one we will still refer to the specialist to check. And very often after we refer to the specialist other than doing basic kidney function and all that, I don’t think we do very much to monitor.” Participant 04_Physician.

### Facilitators to CKD management

#### Knowledge and skills of physicians and patients

Most physicians perceived as being competent on procedural knowledge & having the requisite skills for CKD diagnosis having performed annual CKD screening for albuminuria, calculated eGFR for staging CKD, and using RAS blockers for hypertension control. However, to address gaps in knowledge to effectively manage CKD, many respondents desired CKD education and relevant topics to be ‘taught properly’ and ‘revised frequently’. Several respondents suggested intensive CKD-oriented training while onboarding new medical officers, and continuing medical education activities in the form of renal simulcasts and practical case-based discussions by nephrologists.

Furthermore, all participants expressed an immediate need to improve patient awareness, knowledge and impart self-management skills to facilitate primary care CKD management. Respondents suggested implementation of multi-pronged approaches to patient education and increasing awareness.

“I suppose your usual public education and national programs and all that but I suppose again back to either the clinic staff so it could be the nurses, it could be the doctors, it could be just pamphlets that we give them to read and so on. I think awareness in many forms, I think it is a multi-form approach, you cannot rely on one particular path to improve awareness for CKD.” Participant 08_Physician.

#### Professional role and identity & beliefs about consequences

With a strong sense of professional identity as the ‘key caregiver’ for patients many primary care physicians strongly believed they could manage early-stage CKD. However, they sought more clarity on their role in the CKD care pathway and ‘categorizing patients who need to be cared for at primary care from the SOC’. However, nephrologists expressed the need for mutual discussions to set agreeable guidelines for shared care of patients with more advanced CKD. Importantly, nephrologists viewed the role of family physician clinic managed by physicians trained in family medicine enabling better rapport and facilitating continuity of care for CKD.

#### Belief about capabilities & optimism

Many physicians were comfortable, optimistic, and confident managing early CKD in primary care. But their self-rated confidence was variable with senior physicians perceiving greater confidence compared to junior physicians. A nephrologists concurred that physicians were confident and capable of achieving clinical treatment goals to mitigate CKD risks.

“To be fair they (primary care providers) are pretty good at handling all these diabetic control and things like that, they are in fact quite apt at doing all these things, adjustment things. You just need to give them that that confidence, that they currently they are going on the right track, that they will go and do it properly” Participant 18_Nephrologist.

Few participants stressed the need to cultivate ‘activated and informed patients’ to improve their self-efficacy for chronic disease management. Motivational techniques were regarded as useful, to help delve deeper and understand root cause for non-adherence and conveying messages across in a manner to reassure and reduce patients’ anxiety associated with CKD diagnosis. In all, patient activation and motivational counseling were viewed as patient-oriented facilitators.

#### Intentions and goals

Intention and goals of CKD management were apparent with HCPs unequivocally focusing on risk factor management by controlling diabetes, hypertension, and proteinuria and managing CKD progression. A vast majority of participants advised on smoking cessation, counselled patients on avoidance of non-steroidal anti-inflammatory drugs (NSAIDs), and consciously reviewed medications to adjust to safe renal doses to prevent deterioration in kidney function.

#### Memory attention and decision process

Respondents acknowledged the need for supporting decision-making for nephrology referrals. The physicians preferred more stringent pre-defined criteria for referrals, ensuring early nephrologist review, aiming for appointments within 1 to 2 weeks. Notably, respondents suggested adopting risk-based referral to expedite appointments for at-risk patients and right-site low-risk patients to the polyclinics. Nephrologists supported using the kidney failure risk equation (KFRE) in primary care as a key medical decision-making tool to guide appropriate specialist referrals.

“Perhaps you know we should start maybe in the polyclinic as well, may be to give them a guided kind of decision-making as to that this is the right time or this patient is like this remain like this for next ten years or next two years, so don’t refer so early” Participant 20_Nephrologist.

#### Environmental context and resources

Most participants discussed the utility of technological dashboards to trend creatinine levels and incorporation of automated eGFR into the electronic medical record (EMR) extremely valuable for CKD management. Respondents desired reminders to order kidney tests and alerts for rising creatinine levels to support CKD management. Overall, utilizing technological infrastructure, and clinical-decision support tools enabled appropriate identification, staging, and tracking progression in CKD.

“What has been helpful in recent times was the feature where we could draw for them the creatinine levels from the electronic medical record (EMR) system, so that was really a very good feature. I’ll just put it in and it (EMR system) calculates the eGFR, and even tells you the stage of the disease, so it was an excellent feature”. Participant 10_Physician.

Nevertheless, most physicians did not favor automated referrals based on a single measure of eGFR as they believed clinical correlation was necessary prior to referral. Nephrologists concurred that although patients might fit into the referral criteria based on low eGFR, a physician input was important to assess the patient more holistically prior to referrals.

Considering the time constraints and communication barriers at primary consultation, participants recognized the need for organizational and practice changes, and adoption of team-based care. Many participants underscored the importance of having nurses and dieticians in the team to support counseling in view of the short time available at consults. Many participants suggested engaging multidisciplinary teams comprising nurses, pharmacists, and dieticians in care of patients with poorly controlled chronic disease. Moreover, physicians and nurses perceived the need for personalized motivational counselling, self-management support in terms of dietary advice and reconciliation of medications for complex multi-morbid patients. Thus, team-based care viewed important in CKD management.

“Team-based care is quite important for the patient with poorly-controlled disease, for them to know about their disease process (progression).” Participant 12_Nurse.

In addition, many respondents described collaboration and co-management between primary care and specialists to improve care coordination for CKD. Suggestions include shared-care programs with common platforms where patients visit the specialists annually and primary care every three months, akin to the congestive cardiac failure (CCF) shared-care model.

#### Reinforcement & behavioral regulation

Physicians emphasized the desire for ‘guidelines’, ‘handy reference’ in the form of protocols with clear framework and structured CKD care pathways to reinforce clinician behavior to enable optimal CKD management. They also complemented the new publicly funded initiatives for detection of CKD like the Holistic Approach in Lowering and Tracking Chronic Kidney Disease (HALT-CKD) for timely detection and tracking of CKD.

“This program, called the HALT CKD program, starts with the polyclinic referrals, we make them meet a certain set of criteria like when you refer you know, and you have this patient can you please make sure that they are on an ACE-inhibitor on this particular medicine, can you please make sure blood pressure has reached the target you know.” Participant 20_Nephrologist.

The summary of themes with illustrative quotes for facilitators of and barriers to CKD management is presented in Table 2 and Table 3 respectively.

**Table 2:** Summary of themes and illustrative quotes for barriers to CKD management in primary care

TDF Domains	Themes -Barriers	Illustrative quotes
**Knowledge & Skills**	Knowledge and practice gaps among junior doctors	*“I must say that within the polyclinic setting, we have not been paying that much attention in terms of treatment of CKD, although we know the stages. I believe that knowledge gap may also play a role for lot of the physicians, because we have been actually driving more towards like management of chronic illnesses, even if you look at our doctor’s guidebook it doesn’t really talk much about the CKD management, which is from the specialist clinic.”* Participant 05_Physician *“When I was a junior doctor, I was not very good at CKD because it was not very well taught.”* Participant 09_Physician
	Lack of awareness and self-management skills among patients	*“They come in every time, frequently come in (over) little thing(s), they will come in and see us, you know. I think their health knowledge is not so good for Singaporeans. You know, even for diet (and) all these (things), they can be taking a lot of things, the rich (people) can be very unhealthy; the poor one(s) can be taking something that is not (a) balanced diet.”* Participant 12_Nurse *“We are struggling with a large group of patients with all sorts of problems, like, from poor control, to perhaps symptoms of CKD, to smoking (et cetera), so it’s like (a) whole package right, from either the literacy and their difficulties in reality (et cetera) (that) they are not able to activate themselves to change behaviour, so I think that is really more of the issue rather than communicating the diagnosis.!”* Participant 10_Physician
**Professional role and identity & Beliefs about consequences**	Competing priorities in primary care, CKD less attention by healthcare providers	*I think we cannot generalize this because it depends during the consultation, if there is something that is pressing say for example high blood pressure hypertension and CKD, if that visit itself the blood pressure is high obviously that is going to my priority. But given that, if everything is stable then I think they should be given equal weightage in terms of attention. It is just that we don’t routinely look into the CKD and our knowledge on treating patients with CKD is really lacking. So therefore, currently we give very little attention to CKD*. Participant 05_Physician “*CKD is currently not viewed in the same league as say diabetes, stroke and heart attacks okay. So, barriers would be whether the providers themselves have equal like, do they view CKD as an equally important condition to treat. I think that’s quite fundamental”* Participant 10_Physician
**Beliefs about capabilities & Optimism**	Apprehensions and discomfort in managing CKD complications	*“I think the anaemia ones, anything beyond that what we can give, I think it is hard for us to manage. If let’s say, they already on the maximum iron that we can give here.”* Participant 02_Physician *“Because I mean, lot of times we refer on and we forget about the other things to monitor, so I feel that prevalent problem in our practice is we don’t like monitoring of anaemia, monitoring of calcium level all these and monitoring of bicarb levels we don’t do it very frequently”.* Participant 04_Physician
**Intentions & Goal**	Intention to convey CKD diagnosis in simple terms -ineffective disclosure	*“I will just say in general because I don’t find that if they know the stage, they (patients) will really understand or appreciate it. In the more serious maybe they start having more of the latest stages I will tell them, your kidney is so much damaged now like 60% or 70 just to kind of give them an idea of how bad it is*” Participant 07_Physician *“Number one, they don’t see the reason why they have been referred. Most of the time in CKD 3b even 4 they are fairly asymptomatic right, so they don’t see the reason, they don’t feel unwell. It’s not like derm (skin) we can see is itchy or they would go and see the pathologist, if it’s itchy it bothers them. CKD is asymptomatic, so a lot of times people don’t see the reason for coming as I mentioned to you. So, if it’s not well communicated to them, they don’t understand why they are here (at specialist clinic)”* Participant 19_Nephrologist
	Lack of communication- patients don’t see the reason to visit specialists	*“So [patients] don’t really see the point of seeing renal, because sometimes when they see renal. Renal might just order a bunch of blood for them and they don’t really understand the need for it and what’s the implication of the bloods and don’t really see anything been done for them.”* Participant 02_Physician *“They are aware sometimes; they say they have a little bit of protein in urine, sometimes a little bit of blood. But that’s nothing, nobody tells me (the patient) that it’s dangerous, doesn’t mean that I have a kidney disease. They (patients) don’t know what kidney disease is…. Then they (patient) come to see us, we tell them your kidney is 50% gone (failed) you know, they say hah!! That’s the thing, so, this is a group of people, so we do feel sometimes very frustrated for those patients who don’t know why they are coming for, and on the ground the polyclinic GP don’t explain to them before they come.* Participant 20_Nephrologist
**Memory attention and decision process**	Limitations in decision making for nephrology referrals	*“It’s not so easy also, even though we have some doubts regarding that, is there something we can hence do for the patient, or whether he needs some kind of specialist opinion (and therefore) referring is the best thing.”* Participant 15_Physician “*With nephrotic-range proteinuria, yes, we would (refer), or basically just an increasing trend of creatinine or a reducing trend of eGFR at a slightly fast rate—I don’t really have a figure, it’s just like a feel when I look at the numbers—so those (cases) will be the conscious decision whether a renal physician (should) already be on-board. So, and that would be where we do well and, I think, where we don’t do so well.”* Participant 10_Physician
**Environmental context and resources**	Short consultation time and load	*“Of course, time can be one of the restrictions definitely, because you have to see a lot of patients here, and you hardly give more than five or ten minutes to each patient. And [you are] diagnosing CKD in patients who have never had any problem in their life, this can be challenging at that time because of the time restriction. But still, like if you see high creatinine levels, something is abnormal, you can still ask the patient to repeat it again, or if really, we are not sure, we you refer to the hospital. But of course, time is one of the factors here*.*”* Participant 15_Physician *“I think time, consult time is always a big thing and you need time to explain and do things.”* Participant 08_Physician
	Suboptimal care co-ordination and pathways	Long waiting time for specialist appointment “*I guess in the past it used to be the long waiting time, like in the past it was more than six months, in fact even once it was like a year or something. But I think recently it is bit better and I think what would be beneficial”* Participant 09_Physician *“Unfortunately, our referrals dates are 4 to 6 months away. So sometimes when you are already about 35 eGFR or whatever we just get the date first.”* Participant *08*_Physician Fragmentation of care *“But like I said if they are seeing them only at *ad hoc*, you see them one day, then the other you see somebody else, then that rapport is never built, and it’s very difficult to break all these news to them you know.”* Participant 18_Nephrologist *“Not always seeing the same doctor. Something that they’ve told me before. So, some of them do actually have the same doctor, and those tend to be, they seem happier. Some of them say that every time they come, they see a different doctor so they feel that the doctor may not actually know what’s happening to them.”* Participant 17_Nephrologist Obscure medical information exchange and communication between primary care and specialists *“We have a short- and long-term concerns for our own documentation, then in the specialist we can see their documentation, but the patient with CKD will have lot of other things and have lot of appointments. So, it just gets lost inside and we can’t rely on patients to always remember to bring their memo on hand, they will misplace… because sometimes you have to trouble shoot tons of tracking notes before you find the correct correspondence for the one that you referred for.”* Participant 09_Physician *“So yes, it can be mixed for example you unless you put in the long-term concerns and yes then it will be (available), you would see that, otherwise you can’t miss the previous.”* Participant 03_Physician
	Lack of resources- dieticians, tests, medications	*“I think to manage in primary care can sometimes be, difficult because we don’t have access to all of the test and may be not all the medications that patients can potentially have their choices too*” Participant 03_Physician *“I mean so subsequent later stage of CKD, they need more monitoring like calcium levels or may be the bicarbonate levels which we don’t normally very routinely do here. May be not as frequent it should be done.”* Participant 04_Physician
**Reinforcement & Behavioral regulation**	Believe primary care not doing enough for CKD	“*So, in the SOC’s I am sure, for patients with CKD, they will be screening regularly for example, full blood count because anaemia is one of the complications and they also might be doing things like phosphate levels, calcium levels, they might start patients on calcium pills. So, all this, if you ask me, I think we never do any of this for our patients. Because by certain stage we would have sense it that “Ok this patient is bad enough to refer to the SOC, so from the point we diagnose someone with CKD till the point that we think that the patient is bad enough to refer SOC, we almost never do anything.* Participant 05_Physician *“Every visit, we are saying the same things in primary care, so I think our frustration is that we KNOW all these things, but how come sometimes we just can’t seem to be moving on with the patient, we’re just always stuck in this circle of like, “I know all these, but I can’t do that.” So, I think that is like one of the key things that will change how we deliver care. How do we change this circle of things of just saying things happily [laughs] again and again? It’s not like nobody knows. We all know it but we just can’t seem to get anywhere.* Participant 10_Physician

**Table 3:** Summary of themes and illustrative quotes for facilitators to CKD management in primary care

TDF Domains	Themes -Facilitators	Illustrative quotes
**Knowledge & Skills**	Competent procedural knowledge & skills for diagnosing CKD	*“Let’s say I meet the new patient who has some risk factors and then I will order a renal panel at that point of time. Depending on the results of the renal panel if there is a decrease in eGFR, then I could basically, check for any previous readings of eGFR. If it is more than 3 months apart according to the KDIGO is already considered CKD, then if the eGFR is normal, somehow the patients have some structural or other functional abnormalities like protein urea can also qualify for CKD.”* Participant 06_Physician *“So, you do it based on the blood test, so, (things like) renal panel, eGFR (estimated Glomerular Filtration Rate) and whether they have any other co-existing disease that can lead to CKD. I think I will more rely on the SCM- Lab test results, where I can actually see the results and decide on myself whether or not it is la. For every patient I really look through the panel test anyway and even for patients they are not here for panels, I will still at the previous panel for any chronic patient, I mean we are talking about the chronic care la.”* Participant 02_Physician
	CKD education and training for new medical officers	*“I mean when I was a junior doctor, I was not very good at CKD because it was not very well taught. So that’s why I was thinking like if there were more teaching sessions and case examples to show the use of certain facilities.”* Participant 09_Physician *“Including some time for some renal teaching during the induction, so that before the MO (Medical Officers) comes in, they know a bit of information”.* Participant 14*_*_Physician *“But I think may be CKD, needs to be properly taught to the health professionals and probably need to be revised quite frequently”* Participant 04_Physician
	Awareness, education and improving self-management skills for patients	“*Actually, education. Health education for our patient(s) disease, like more pertaining to kidney disease, what (they) should do, what to take and then, how to prevent hypertension (and) all these (diseases). Educating patient (is) also important.”* Participant 12_Nurse “*The first thing is the awareness. The first thing is awareness, like, we should educate more of the patients (on) what they have (for their) underlying condition, and what they should do to keep health in the first place.”* Participant 15_Physician
**Professional role and identity & Beliefs about consequences**	Improving clarity on the role of primary care physician in CKD care pathway	“*May be more communication between the specialist and the primary care, so that we can clearly define the stage of CKD, that can comfortably managed in the polyclinic setting and those patients who need to be cared for at the SOCs”* Participant 05_Physician “*Maybe also clarity of roles, because at this point, I’m not sure when the kidney doctors wants to see the cases because the climate has changed (and) there’s probably quite A LOT OF CKD patients around. So, they may be swamped with CKD (Stage) 3 and perhaps then, how do we manage some of the CKD (patients)—which I think we CAN*” Participant 10_Physician
	Family physician clinic-enabling rapport and continuity of care	*“I do have a few but mainly at the family physician clinic (FPC) but FPC is a slightly different clinic as you see more complex patients’ longer consults, those you see them back quite frequently so that one in terms of continuity of care is not an issue, usually there will be some correspondence with the specialist and send back to you, that one not a problem*.” Participant 09_Physician *“When I run the family clinic they (patients) have seen me for about 5 to 10 years. So they are quite accepting to it but in general clinic I do have hurdles sometimes because they may or may not agree. In this case we tell them, “Ok you think about it, next visit we will do it”.* Participant 08_Physician “*I mean I love that chronic system, but I am not sure of what the name of that system is or program where they usually see one doctor, and then because, I can tell right with the notes, I look at the prescription it is always the same polyclinic doctor who is prescribing and then that kind of system I feel that patient is actually is more aware of his condition, there is a lot more better communication, usually the patient groups who actually know, when I talk to them I feel that they understand a bit more. And the polyclinic system has done very well for those patients. And as I mentioned just now it makes it easier if I do want someone or somebody to communicate with because I know who to look for.”* Participant 19_Nephrologist
**Beliefs about capabilities & Optimism**	Confident managing CKD in primary care	*“So, of course, ours is (that we are) confident about blood targets, because you code the proteinuria, you look for proteinuria to see how’s the situation, monitor through the trends, and you are looking specifically at medications like ACEs (angiotensin-converting-enzyme* inhibitor)*, ARBs (Angiotensin II receptor blockers), ensure they are on board, (that) they are maximized to whatever the patient can tolerate; (and) LDL glycaemic target(s), so I think that these are very fundamental, and I think primary care is actually quite confident in doing that.”* Participant 10_Physician *“I think it (confidence) is sufficient. I think the education we get and the guidelines we get from our management, generally enough for us to manage them (patients with CKD) comfortably*.” Participant 08_Physician
	Patient activation through motivational interviewing	*“So in order to motivate patients, I think like I mentioned before, you know about motivation interview right? It is how you put the message across the patients, how do you want to educate your patients, you must do this, you must do that, that patients will not follow. You must tell them, “Sir you only see me three monthly four monthly, the optimal of your health is in your hands, you really have to take care of yourself. You must be responsible and you don’t want it (disease) to be getting worse and worse, stroke coming in, heart attack coming in kidney failure coming in. So I think it is good to find out from patients their main issue, the root cause of non-compliance. It is how you put the message across the patients, how do you want to educate your patient”* Participant 16_Nurse “*Yes, the group still willing to take medications or to protect their body, when you just highlight to them what is the purpose of the medications they will take it. But, the group not interested in their health, you need to follow other techniques to motivate them and what is the value actually. Basically, you need activated you need an informed patient and you also need an activated team. You cannot have none of each or one of two. If both activated, then you can actually have a better management of the chronic disease”* Participant 06_Physician
**Intentions & Goal**	Deliberate risk factor management to delay CKD progression	*“I would just manage the patient based on making sure that they don’t progress to Stage 4 or Stage 5, by managing their chronic disease, like OTHER chronic disease like Diabetes, Hypertension, (to) the best (ability) I can. Then, (I) explain to the patient, you know, that other causes that can make kidney functions go worse”* Participant 01_Physician *“I think as a family physician just to recognize those who are at risk of developing CKD and also prevent them to get that. Secondly, if they do have CKD prevent them from worsening control of chronic diseases.”* Participant 04_Physician
**Memory attention and decision process**	Facilitating decision making for nephrologist referral – Fast track referral and KFRE	“*You need to in-build this into the whole system because now you know the internet is all cut off you can calculate it by yourself on your laptop and on your hand phone, but how difficult and how challenging that is, if you can ultimately produce that number and then it’ll be easier thing for them to refer to. KFRE I think is a good way to go, and that’s what I was thinking for my discharge criteria also. If patients they can be safely discharged and they are low risk of progression. So yes, KFRE is a good way.”* Participant 18_Nephrologist *“I think using stricter guidelines, so those who need it, will get it first. So if you could have, for this patient may be a fast track queue or normal queue, but they need to do the blood test and it is a quick one and non-fasting we try to keep it in the afternoon, so they don’t queue in the morning fasting bloods, but sometimes that is also difficult but fast track will make a difference, then they should be more willing to do this.”* Participant 08_Physician *“Fast track referrals are for patients’ that (aa) polyclinic deems as need to be seen soon. So, fast track I don’t think we have any issue with fast tracking them. So they have fairly early appointments, so the fast track ones are seen quite quickly or you know it’s not going to be like a few months wait, definitely within 1–2 weeks most of the time.”* Participant 19_Nephrologist
**Environmental context and resources**	Utilizing clinical decision support tools for CKD care	*“So, it is good that we have a built in calculator now that tells us what stage of CKD the patient is at, that helps to facilitate the identification of the patient. Secondly, I think it is good that now the SCM system actually sort of provide us with the criteria when we are supposed to refer. So that is another good point, I think those are the facilitators.”* Participant 05_Physician *“Recently Dr XXXXX in SHP also started this calculator thing so it can also be used and he also built in KDIGO score into this calculator. I think it is quite good, the one most convenient one is the Clin doc one because once you click the thing it (eGFR) will be auto calculated. So I felt it’s very useful.”* Participant 06_Physician
	Multidisciplinary team-based delivery of care	*“Team-based care. Like, we have team-based care whereby the MDT (Multidisciplinary Team) cases will see the nurse counsellors first before they see the doctor. All these will help with the control of the patient(s), counsel the patients on their diet (and) all these. Team-based care is quite important for the patient with poorly-controlled disease, for them to know about their disease process (progression).”* Participant 12_Nurse “*So your pharmacist will be advising them on medications and side-effects and nurse will be advising them on diet and medication timings and all that. The doctor will be advising on the future on the prognosis and I think there is a place for team-based care. I guess all these things are already in place but if you want to improve by getting somebody to counsel patients with CKD and sort of follow them up from the moment they start the counselling to see whether there is change in lifestyle and so on.”* Participant 08_Physician
**Reinforcement & Behavioral regulation**	Reinforcing provider knowledge and structured CKD care pathways	*“So I mean if there is education on the implementation part of it would be good. I mean, the CKD part, the content generally you can read up on it, e.g., the guidelines, how to manage? What are the complications to look out for? I think in terms of what can be done in the polyclinic, they (junior doctors) may not know we can refer for BMD. They may not know we have fast track referral criteria. So I guess, may be that part may be highlighted to the junior doctors so that they can make use of the existing services.”* Participant 09_Physician *“So, if you have a structured pathway built into this that might be, like we know the we need to order the panel every six months, so if you can reinforce to them that for our doctors, this is what should be done for CKD then I think people will follow and can benefit the patients.”* Participant 06_Physician *“Definitely (clear guidelines) would be good. I mean, we have all these things on the board, to tell us, like, about guidelines for this, guidelines for that, so if there is ready information on CKD, (to) just look up and follow those guidelines would be easier, like a flowchart or something like that.”* Participant 14_Physician
	Implementation of programs for timely detection and tracking within primary care	“*HALT-CKD is probably very useful and necessary because there is significant burden of CKD in our patients. I do have renal physician friends and I am sure they are drowning in such patients, and I am sure there are not enough dialysis centers. It is something we address but I think the CKD is caused by the chronic diseases, DM which we are attacking, and hypertension. So if you ask me, I think that’s also area that need a lot more attention because we can prevent the CKD in the first place, we don’t have to end up like trying to treat it and often it still gets worse with everything”* Participant 07_Physician “*This program we have a certain ministry, called the HALT CKD program, starts with the polyclinic referrals, we make them meet a certain set of criteria like when you refer you know, and you have this patient can you please make sure that they are on an ACE-inhibitor on this particular medicine, can you please make sure blood pressure has reached the target you know. Cannot just refer just because there’s CKD. But before you refer can you do something first right. Optimize the blood pressure control, optimize the glucose control, making sure that they are taking their medicine *etc*. *etc.*… So, that’s step 1”* Participant 20_Nephrologist

## Discussion

HCPs in primary care are the first point of contact for most patients with CKD, well-positioned to institute therapy to prevent ESKD and other complications and arrange timely referrals to nephrologists. Our study explored barriers and facilitators to CKD management in primary care, taking into account the perspectives of HCPs, including physicians (predominantly primary care physicians and nephrologists), nurses, and pharmacists involved in the continuum of care for CKD. Our findings demonstrate that HCPs believe that CKD remains neglected in primary care due to inadequacy in resource allocation, diagnosis disclosure, consultation time, physician–patient communication, lack of patients' awareness and self-management skills, and the referral clarity to nephrologists. Nevertheless, essential enablers to CKD management include CKD specific-training of primary care physicians, structured CKD-care pathways, multidisciplinary team-based care, and transparent criteria for nephrology referrals. Of note, physicians expressed willingness to manage early-stage CKD in the primary care setting, leveraging on a collaborative CKD care model with the nephrologists. Thus, our findings provide valuable insights for improving CKD care delivery in Singapore's primary care setting, which may also be relevant to other countries.

Our findings of barriers of knowledge and practice gaps among physicians, competing priorities with less attention to CKD in primary care, and ineffective physician–patient communication of CKD diagnosis are aligned with findings from previous studies [18–20]. A strong need for continuous professional education in CKD management was highlighted as key developmental area. Of note, mentorship programs with nephrologists, like those in Canada [21] could offer benefit. Alongside, it is vital to address barriers of patient-physician communication. Understanding key competencies and creating a competency-based curriculum to equip clinicians with skills for communication and patient engagement around CKD diagnosis [22] are essential to facilitate CKD management.

HCPs highlighted short consultation time, suboptimal care coordination and fragmentation of care as major barriers to CKD management which have been reported previously from several regions globally [23–25]. In the background of such health system and contextual barriers, HCPs desired team-based care for CKD concurrent with earlier studies [10, 20, 26]. Primary care team-based models may potentially address barriers like time and workload, patient-physician communication, and elevate CKD literacy and self-care skills for patients. Team-based model has shown to be effective in the management of chronic diseases such as hypertension, diabetes mellitus, and depression [27–29]. Similar collaborative team-based care models with physicians, nurses and dieticians working synergistically has enhanced both guideline-based CKD care delivery [30] and associated patient-provider communication.

Clear and concise guidelines with roadmap for care and referral pathways have been previously identified as enablers of CKD management in primary care [10, 31]. Although quality resources and tools exist, guidance on referral criteria is not adhered to stringently due to lack of awareness, comprehension, and challenges in uptake and utilization jeopardizing timely nephology referrals. Of note, although not currently implemented, HCPs supported the use of KFRE in practice to facilitate referral to the nephrologists. Literature has revealed that timely outpatient nephrology referral slows disease progression, reduces hospital admissions, reduces total treatment costs, and improves survival in patients with CKD [32]. Prioritizing referrals based on instrument such as KFRE, which has been validated in local primary care clinics [33] are considered valuable for risk assessment and decision-making. Establishing wait-time benchmarks and risk-based triage in Canada have reduced referral delay, improved access and provided targeted care for patients with highest risk of progression to ESKD [34–36]. Additionally, clinical decision-support tools embedded in a common EMR could facilitate primary care-specialist communication, case review and shared decision-making in siting patients with CKD across the primary-tertiary care interface [37, 38].

In addition, patient-level lack of knowledge, awareness, and poor self-management skills perceived by the HCPs are commonly reported in literature and by us [10, 25, 39]. Lack of awareness and knowledge on their chronic condition often limits individuals to engage in desired self-management behaviours as recommended by physicians. Multipronged approach to patient education, and increasing patient engagement have been recommended as useful strategies for improving self-management of CKD [11].

There are clear challenges to CKD management that are persistent in primary care. Optimal CKD intervention strategies should aim to address the multiple impediments and leverage on the opportunities. Structured CKD programs with multicomponent interventions need evaluation in Singapore and similar countries.

### Strengths and limitations

The study is likely among the first qualitative studies to explore HCPs’ perceptions on the barriers and facilitators to CKD management in a developed primary care community. Including different cadres of healthcare professionals in primary care and combining nephrologists' viewpoints contributed to a fuller understanding of CKD management in the care continuum. The participants had no personal or professional relationship with the interviewers, and this allowed them to express their opinions openly. We used a sound conceptual TDF framework, [13] to delve deep and understand the complex and interlinked barriers and facilitators in CKD management from different stakeholders’ perspective. Our findings are relevant to other primary HCPs with similar healthcare system.

Our study has its limitations. We did not include private general practitioner clinics. However, the majority of patients with chronic disease including CKD, regardless of their socioeconomic backgrounds are treated at public primary care establishments in Singapore. Furthermore, participant responses might have been influenced by the TDF-based questions, and the study was not designed for in-depth assessment of adequacy of physician knowledge and skills regarding management CKD, although questions in the topic guide covered broad principles of CKD management as per international clinical practice guidelines. Despite efforts to recruit a balanced sample, more female participants which might introduce bias.

## Conclusions

CKD is a rising healthcare challenge in Singapore and globally. Primary care system provides the greatest opportunity to intervene and achieve better clinical outcomes in CKD. We identified potentially modifiable barriers and facilitators to optimize  management of CKD in primary care from HCP’s perspectives. Foremost, CKD deserves greater priority from HCPs and health planners in primary care. Besides, gaps in care coordination, patient-physician communication and provider knowledge need urgent attention. Moving forward, collaborative team-based care models using multifaceted strategies for structured CKD management, risk stratification to prioritize nephrology referrals using validated instrument, coupled with CKD training for all primary care physicians and self-management education for patients need implementation and evaluation. Such concerted efforts are likely to improve outcomes of patients with CKD and reduce the ESKD burden.

## Supplementary Information

**Additional file 1.** Consolidated criteria for reporting qualitativestudies (COREQ): 32-item checklist.

## Data Availability

The datasets used and/or analyzed during the current study are available from the corresponding author on reasonable request.

## Abbreviations

CVD: Cardiovascular disease
CKD: Chronic kidney disease
eGFR: Estimated glomerular filtration rate
EMR: Electronic medical records
ESKD: End stage kidney disease
HCPs: Healthcare professionals
KFRE: Kidney failure risk equation
RAS blockers: Renin angiotensin system blockers
SOC: Specialist outpatient clinics
TDF: Theoretical domains framework
